# Supplementary material for: COZOID: contact zone identifier for visual analysis of protein-protein interactions
Source: BMC Bioinformatics. 2018 Apr 6;19:125. doi: 10.1186/s12859-018-2113-6 (PMC5889581; doi:10.1186/s12859-018-2113-6)
Supplement: Supplementary file 2 — Software build. Executable binary file of the software tool. (ZIP 118,784 kb) [file 12859_2018_2113_MOESM2_ESM.zip › cozoid/java/docs/beansbinding-1.2.1-doc.zip!/org/jdesktop/el/MethodExpression.html]

MethodExpression (Beans Binding)


---


|  |  |  |
| --- | --- | --- |
| **Overview** \| **Package** \| **Class** \| **Use** \| **Tree** \| **Deprecated** \| **Index** \| **Help** | |  |
| **PREV CLASS**   **NEXT CLASS** | **FRAMES**    **NO FRAMES**     **All Classes** |  |
| SUMMARY: NESTED \| FIELD \| CONSTR \| METHOD | DETAIL: FIELD \| CONSTR \| METHOD |  |


---


## org.jdesktop.el Class MethodExpression

```
java.lang.Object
  org.jdesktop.el.Expression
      org.jdesktop.el.MethodExpression
```

**All Implemented Interfaces:**: java.io.Serializable

**Direct Known Subclasses:**: MethodExpressionImpl, MethodExpressionLiteral

---

``` public abstract class MethodExpression extends Expression ```

An `Expression` that refers to a method on an object.

The `ExpressionFactory.createMethodExpression(org.jdesktop.el.ELContext, java.lang.String, java.lang.Class , java.lang.Class [])` method
can be used to parse an expression string and return a concrete instance
of `MethodExpression` that encapsulates the parsed expression.
The `FunctionMapper` is used at parse time, not evaluation time,
so one is not needed to evaluate an expression using this class.
However, the `ELContext` is needed at evaluation time.

The `getMethodInfo(org.jdesktop.el.ELContext)` and `invoke(org.jdesktop.el.ELContext, java.lang.Object[])` methods will evaluate the
expression each time they are called. The `ELResolver` in the
`ELContext` is used to resolve the top-level variables and to
determine the behavior of the `.` and `[]`
operators. For any of the two methods, the `ELResolver.getValue(org.jdesktop.el.ELContext, java.lang.Object, java.lang.Object)`
method is used to resolve all properties up to but excluding the last
one. This provides the `base` object on which the method
appears. If the `base` object is null, a
`PropertyNotFoundException` must be thrown.
At the last resolution,
the final `property` is then coerced to a `String`,
which provides the name of the method to be found. A method matching the
name and expected parameters provided at parse time is found and it is
either queried or invoked (depending on the method called on this
`MethodExpression`).

See the notes about comparison, serialization and immutability in
the `Expression` javadocs.

**Since:**
:   JSP 2.1

**See Also:**: `ELResolver`, `Expression`, `ExpressionFactory`, Serialized Form

---

**Nested Class Summary**

**Nested classes/interfaces inherited from class org.jdesktop.el.Expression**

`Expression.ResolvedProperty, Expression.Result`


**Constructor Summary**

`MethodExpression()`


| **Method Summary** | |
| --- | --- |
| `abstract  MethodInfo` | `getMethodInfo(ELContext context)`             Evaluates the expression relative to the provided context, and returns information about the actual referenced method. |
| `abstract  java.lang.Object` | `invoke(ELContext context, java.lang.Object[] params)`             If a String literal is specified as the expression, returns the String literal coerced to the expected return type of the method signature. |

**Methods inherited from class org.jdesktop.el.Expression**

`equals, getExpressionString, hashCode, isLiteralText`

**Methods inherited from class java.lang.Object**

`clone, finalize, getClass, notify, notifyAll, toString, wait, wait, wait`

**Constructor Detail**

### MethodExpression

```
public MethodExpression()
```


**Method Detail**

### getMethodInfo

```
public abstract MethodInfo getMethodInfo(ELContext context)
```

:   Evaluates the expression relative to the provided context, and
    returns information about the actual referenced method.

    :   **Parameters:**: `context` - The context of this evaluation **Returns:**: an instance of `MethodInfo` containing information about the method the expression evaluated to. **Throws:**: `java.lang.NullPointerException` - if context is `null`: `PropertyNotFoundException` - if one of the property resolutions failed because a specified variable or property does not exist or is not readable.: `MethodNotFoundException` - if no suitable method can be found.: `ELException` - if an exception was thrown while performing property or variable resolution. The thrown exception must be included as the cause property of this exception, if available.

---


### invoke

```
public abstract java.lang.Object invoke(ELContext context,
                                        java.lang.Object[] params)
```

:   If a String literal is specified as the expression, returns the
    String literal coerced to the expected return type of the method
    signature. An `ELException` is thrown if
    `expectedReturnType` is void or if the coercion of the String literal
    to the `expectedReturnType` yields an error (see Section "1.16 Type
    Conversion" of the EL specification).
    If not a String literal, evaluates the expression
    relative to the provided context, invokes the method that was
    found using the supplied parameters, and returns the result of
    the method invocation.
    Any parameters passed to this method is ignored if isLiteralText()
    is true.

    :   **Parameters:**: `context` - The context of this evaluation.: `params` - The parameters to pass to the method, or `null` if no parameters. **Returns:**: the result of the method invocation (`null` if the method has a `void` return type). **Throws:**: `java.lang.NullPointerException` - if context is `null`: `PropertyNotFoundException` - if one of the property resolutions failed because a specified variable or property does not exist or is not readable.: `MethodNotFoundException` - if no suitable method can be found.: `ELException` - if a String literal is specified and expectedReturnType of the MethodExpression is void or if the coercion of the String literal to the expectedReturnType yields an error (see Section "1.16 Type Conversion").: `ELException` - if an exception was thrown while performing property or variable resolution. The thrown exception must be included as the cause property of this exception, if available. If the exception thrown is an `InvocationTargetException`, extract its `cause` and pass it to the `ELException` constructor.


---


|  |  |  |
| --- | --- | --- |
| **Overview** \| **Package** \| **Class** \| **Use** \| **Tree** \| **Deprecated** \| **Index** \| **Help** | |  |
| **PREV CLASS**   **NEXT CLASS** | **FRAMES**    **NO FRAMES**     **All Classes** |  |
| SUMMARY: NESTED \| FIELD \| CONSTR \| METHOD | DETAIL: FIELD \| CONSTR \| METHOD |  |


---
